# Emerging methods for prostate cancer imaging: evaluating cancer structure and metabolic alterations more clearly

**DOI:** 10.1002/1878-0261.13071

**Published:** 2021-08-30

**Authors:** Adam Retter, Fiona Gong, Tom Syer, Saurabh Singh, Sola Adeleke, Shonit Punwani

**Affiliations:** ^1^ UCL Centre for Medical Imaging London UK

**Keywords:** biomarker, imaging, metabolism, MRI, PET, prostate cancer

## Abstract

Imaging plays a fundamental role in all aspects of the cancer management pathway. However, conventional imaging techniques are largely reliant on morphological and size descriptors that have well‐known limitations, particularly when considering targeted‐therapy response monitoring. Thus, new imaging methods have been developed to characterise cancer and are now routinely implemented, such as diffusion‐weighted imaging, dynamic contrast enhancement, positron emission technology (PET) and magnetic resonance spectroscopy. However, despite the improvement these techniques have enabled, limitations still remain. Novel imaging methods are now emerging, intent on further interrogating cancers. These techniques are at different stages of maturity along the biomarker pathway and aim to further evaluate the cancer microstructure (vascular, extracellular and restricted diffusion for cytometry in tumours) magnetic resonance imaging (MRI), luminal water fraction imaging] as well as the metabolic alterations associated with cancers (novel PET tracers, hyperpolarised MRI). Finally, the use of machine learning has shown powerful potential applications. By using prostate cancer as an exemplar, this Review aims to showcase these potentially potent imaging techniques and what stage we are at in their application to conventional clinical practice.

## Graphical abstract

Imaging plays a fundamental role in all aspects of cancer management. However, conventional imaging methods are largely reliant on morphological descriptors that have well‐established limitations. Although functional techniques have partially mitigated these shortcomings, they are still imperfect. Recently, potentially potent novel imaging techniques assessing cancer microstructure, metabolism and the use of machine learning have entered the pipeline to conventional application.

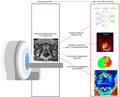

**Abbreviations**
ADC: apparent diffusion coefficient
CAD: computer‐aided diagnosis
CT: computerised tomography
DCE: dynamic contrast enhancement
DL: deep learning
FDG: fludeoxyglucose (^18^F)
LWF: luminal water fraction imaging
ML: machine learning
Mp‐MRI: multiparametric magnetic resonance imaging
PCa: prostate cancer
PET MRI: positron emission technology MRI
VERDICT: vascular, extracellular and restricted diffusion for cytometry in tumours

## 1 Introduction

Imaging is implemented at every stage of the cancer management pathway, from diagnosis, staging, prognostication, surveillance and assessment of treatment response, to detection of complications following treatments and confirming remission [1]. The major advantage of imaging over other diagnostic tests remains its inherent ability to spatially localise disease.

Conventional imaging techniques – radiography, ultrasound (US), computerised tomography (CT) and magnetic resonance imaging (MRI) – rely upon morphological/anatomical features or descriptors in the evaluation of tumours. Features that are described qualitatively and subjectively, such as ‘spiculated’, ‘moderate heterogeneity’ and ‘obscured margins’, can help differentiate benign from malignant pathologies. However, although descriptors can provide an indicator towards malignancy, they are not perfect. For example, size is commonly employed as a classifier of malignancy within lymph nodes; yet, nodes less than a given size threshold may still harbour cancer (indicating limitations of sensitivity) [2, 3], and those larger than the threshold may be enlarged due to alternative aetiology such as infection (indicating limitations of specificity) [4].

Nonetheless, assessment of response to systemic cancer therapy remains contingent on size change, evaluated by response evaluation for solid tumours (RECIST) criteria, updated in 2009 to version 1.1 [5]. This has its limitations; for example, irregular and diffusely infiltrating lesions and lesions that are nonspherical are difficult to consistently measure, and diffuse bone lesions cannot be assessed altogether. Moreover, chemotherapeutic response may not itself cause a change in size and may, in some cases, increase the size of lesions [6]. Concealed responses can also occur, where imaging indicates no alteration in size when, histologically, there has been a complete response [7].

Significant effort is therefore focused on developing new imaging methods that can address these limitations. These methods aim to complement morphological imaging, with assessments of microstructure, function and metabolism – tissue attributes that are commonly altered in the development of cancer.

Within this Review, by using prostate cancer (PCa) MRI‐based imaging as an exemplar, we highlight clinically available cancer imaging methods and showcase novel and emerging imaging methods which use our current clinical imaging infrastructure. Limiting the scope to PCa MRI enables detailed insight that may be transferrable to other cancers. PCa showcases a cancer where investigation with MRI is an extensively studied area, and it exhibits how the integration of researched functional imaging techniques can change the standard of care [8].

## 2 Current role of multiparametric MRI in prostate cancer

The traditional diagnostic pathway was based on a transrectal US‐guided (TRUS) biopsy being offered to all men deemed at risk for PCa. However, high rates of overdiagnosis, missed significant lesions, misclassifications and potentially significant complications have led to the present implementation of multiparametric MRI (Mp‐MRI) [9, 10, 11, 12, 13]. UK National Institute for Health and Care Excellent guidelines, updated on 09/05/2019, recommend that Mp‐MRI be offered as the first‐line investigation technique to people with suspected localised PCa [14]. Furthermore, the European Association of Urology strongly recommend that Mp‐MRI be used before biopsies in biopsy‐naïve men and, when MRIs are positive in this cohort [prostate imaging reporting and data system (PIRADS) ≥ 3], that targeted and systemic biopsies are performed, referring to the ‘MRI pathway’.

Multiparametric‐MRI combines anatomical sequences (T1‐ and T2‐weighted MRI) and at least two functional techniques that are readily clinically available. The added value of these techniques, as evidenced by the Prostate MRI Imaging Study trial, improves the detection of clinically significant disease compared with TRUS biopsies using transperineal template mapping biopsies as the reference standard (sensitivity of 93% vs 48%, negative predictive values 89% vs 74% in individuals with a Gleason score ≥ 4 + 3) [15]. The prospective multicentre PRECISION trial randomly assigned biopsy‐naïve men to either have an Mp‐MRI and a targeted biopsy (only if the MRI was positive) or a TRUS biopsy, without an Mp‐MRI. In the Mp‐MRI group, 28% of men avoided biopsy following a negative MRI and 38% of men that did receive targeted biopsies were diagnosed with clinically significant disease. Comparatively, 26% of men in the TRUS biopsy group were diagnosed with significant disease and more men were diagnosed with insignificant cancers (22% vs 9%) [16]. Similarly, the 4M study showed no difference in significant disease detection between the MRI and TRUS pathways (25% vs 23%); however, performing an MRI avoided biopsies in 49% of the men [17]. However, as shown by the multicentre MRI‐FIRST study, in which patients received both TRUS biopsy and targeted biopsy if they were MRI positive, clinically significant PCa would have been missed in 5.2% of patients had a TRUS biopsy not been performed; thus, the need for TRUS biopsy was not excluded [18].

The functional imaging techniques utilised in the Mp‐MRI pathway can achieve fewer men having biopsies, more significant disease identified and less overdetection of insignificant cancer. Despite the step change in diagnostic performance that Mp‐MRI enables, it is not a perfect test. Significant research efforts focusing on the development of imaging to facilitate simplification, improve diagnostic accuracy and offer potential prognostic application are ongoing.

## 3 Clinically available functional imaging

Developing imaging methods follows a translational pipeline. Many methods emerge and are showcased in preclinical studies [19, 20], however, only a few transition to first‐in‐human proof‐of‐concept studies [21]. These reports generate considerable excitement but seldom address repeatability, and the technique often remains immature, in that it does not have proven clinical value or impact on patient management [22]. Indeed, many of these technologies will never progress further as they are unable to overcome the challenges of becoming a ‘product’ [23, 24]. These challenges include biological, technical, clinical and outcome validation [25]. As such, within this section, we concentrate on techniques that utilise equipment commonly available in the hospital setting – in particular MRI and positron emission tomography (PET) modalities, which can today go beyond anatomical assessment and interrogate the microstructural, functional and/or metabolic properties of cancer.

### 3.1 Diffusion‐weighted MRI

Diffusion‐weighted MRI gives insight into tissue microstructure. In its most commonly implemented form, it interrogates tissue cellularity [26, 27, 28]: diffusion‐weighted imaging (DWI) signal intensity is increased in most cancers due to the hindered translational motion of water molecules caused by high cellularity. The degree to which an image is sensitised to water diffusion is controlled by the operator by means of setting a ‘b‐value’ (commonly set between 0 and 2000 s·mm^−2^); a product of the amplitude, duration and timing of diffusion‐sensitising magnetic field gradients is applied during signal generation.

Whilst DWI images are often visually assessed, the signal intensity of images acquired with increasing b‐values is also exponentially fitted to allow quantitation of the apparent diffusion coefficient (ADC). ADC values are typically lower in malignant tissue when compared to surrounding normal tissue. In clinical practice, DWI has shown value in tumour detection, staging and treatment follow‐up (including immune therapies) [29, 30]. Furthermore, the ADC, and the ADC ratio of the lesion compared to the neighbouring tissue, is able to predict the Gleason grading of PCa lesions [31].

### 3.2 Dynamic contrast‐enhanced MRI

Dynamic contrast enhancement‐MRI (DCE‐MRI) enables the assessment of microvasculature of the lesion; for cancer, it enables assessment of neovascularisation [32]. Tumour vasculature is characterised by a chaotic structure, a high number of dysfunctional vessels, complex branching patterns, abnormal permeability and nonuniform vascular densities, together resulting in blood flow that is spatially and temporally heterogeneous [33]. DCE‐MRI evaluates this by using contrast agents that cause signal changes dependent on changes in blood flow, capillary density, permeability and extravascular‐space volume induced by cancer. Fast T1‐weighted MRI sequences are used to acquire repeated images depicting the signal changes that occur with the arrival and washout of contrast within a particular region, with a rapid enhancement and washout being typical of most cancers. DCE images can be visually assessed and analysed through signal‐intensity time curves or through models which quantify pharmacokinetic parameters (e.g. *K^trans^*, *K*_ep_, *v_e_*; see below).

Qualitative assessment of DCE images relies upon localising regions of early enhancement and washout when compared to surrounding normal tissue. Signal‐intensity time curves are generated through region of interest (ROI) placement and temporal extraction of mean ROI signal across sequential T1‐weighted acquisitions. Time to start of enhancement, time to peak enhancement, maximum enhancement, slope of enhancement and area under the curve metrics can be derived and related to tissue vascular structure [34, 35]. The most commonly used pharmacokinetic model (Tofts model) fits the signal‐intensity time curves to a one‐compartment model, allowing the calculation of extravascular extracellular space volume (*v_e_*) and a transfer constant (*K^trans^*, a measure of proportionality of distribution of contrast between the vascular and extravascular extracellular space). The vascular compartment volume is ignored as, for most cases, it is a relatively small fraction (1–10%) of the tissue [36, 37, 38]. Quantitative DCE‐MRI is used within clinical trials as an endpoint for drug efficacy [21]; curve‐shape assessment is routinely utilised clinically for breast imaging [39]; and visual assessment is recommended for the evaluation of PCa [40, 41].

### 3.3 Magnetic resonance spectroscopy

Clinically applied magnetic resonance spectroscopy (MRS) techniques are not strictly an emerging entity and have been trialled since the availability of clinical‐grade MRI scanners. They enable the generation and separation of MR signals from ^1^H nuclei from different metabolites. Differences in the electron shielding of the ^1^H nucleus between metabolites cause distinct frequencies of signal for individual metabolites [42]. However, the concentration of metabolites is many magnitudes smaller than the concentration of water within the body, and therefore, high‐field magnets, long imaging times and a nonlocalised signal are commonly required to achieve metabolite signal‐to‐noise ratios sufficient for interpretation. These limitations, together with the limited availability of expertise to set up and process spectroscopy data, have meant that MRS is not widely employed in the clinical imaging of cancer. Nonetheless, metabolites in the millimolar range (including choline, creatine, lactate and citrate) are detectable and have been clinically evaluated within limited cancer applications. For instance, choline, a metabolic marker of cell membrane synthesis and repair found in more rapidly proliferating cells, has been demonstrated to be a characteristic signature of more aggressive tumours [43]. Although no longer part of the standard PCa multiparametric imaging protocol, when used in combination with T2‐MRI and DCE‐MRI, sensitivities for tumour detection are higher [44].

### 3.4 Positron emission technology

Positron emission technology is a molecular imaging technique that can provide high sensitivity through use of positron emitters to label key molecules that are intravenously injected and their distribution and uptake imaged to provide insight into metabolic changes associated with cancer. Whilst PET scans can be viewed alone, because of their limited morphological detail hybrid instruments are commonly used to combine anatomical CT or MRI images with PET metabolic information [45, 46].

## 4 Novel imaging methods

Despite the now almost routine use of the techniques discussed above [e.g. Mp‐MRI for PCa using DWI and DCE imaging [47, 48], staging of breast cancer using fludeoxyglucose (^18^F) (FDG)]‐PET [49] or evaluation of brain tumours using MRS) [50, 51], the challenges of limited sensitivity, specificity, tumour characterisation and prognostication have not been fully addressed. Therefore, there is still considerable effort directed towards developing new and improved imaging methods. This section outlines a selection of novel approaches that have demonstrated promising initial results.

### 4.1 Machine learning applied to currently available cancer imaging techniques

The general supposition of machine learning (ML), when applied to radiology, is that images contain information on pathology which can be extracted by a computer algorithm with performance either as good as or better than a human. A set of images with a known ground truth (training set) are used to train an ML system so that, when new images (test set) are shown to the system, it can make a prediction based on its previous experience. This approach has been very successful for natural image analysis, even surpassing human ability in some tasks [52]. These same methods are being developed for medical imaging to improve the efficacy and efficiency of cancer detection and characterisation. ML can utilise quantitative metrics, referred to as radiomic features, such as shape, uptake values and other features, including second‐order features (texture) [53].

There are two commonly investigated ML methods for medical image analysis: classical ML and deep learning (DL). Classical ML usually requires manual segmentation of lesions and extraction of quantitative imaging features such as shape, volume, histogram and texture from a training set. Those features are used to train the ML algorithm, which then uses statistical analysis to identify the optimal relationship between the imaging features in order to predict the desired outcome. There are multiple types of algorithms, such as decision trees [54], support vector machines and naïve Bayes, each of which may be better suited to individual tasks.

**Fig. 1:**
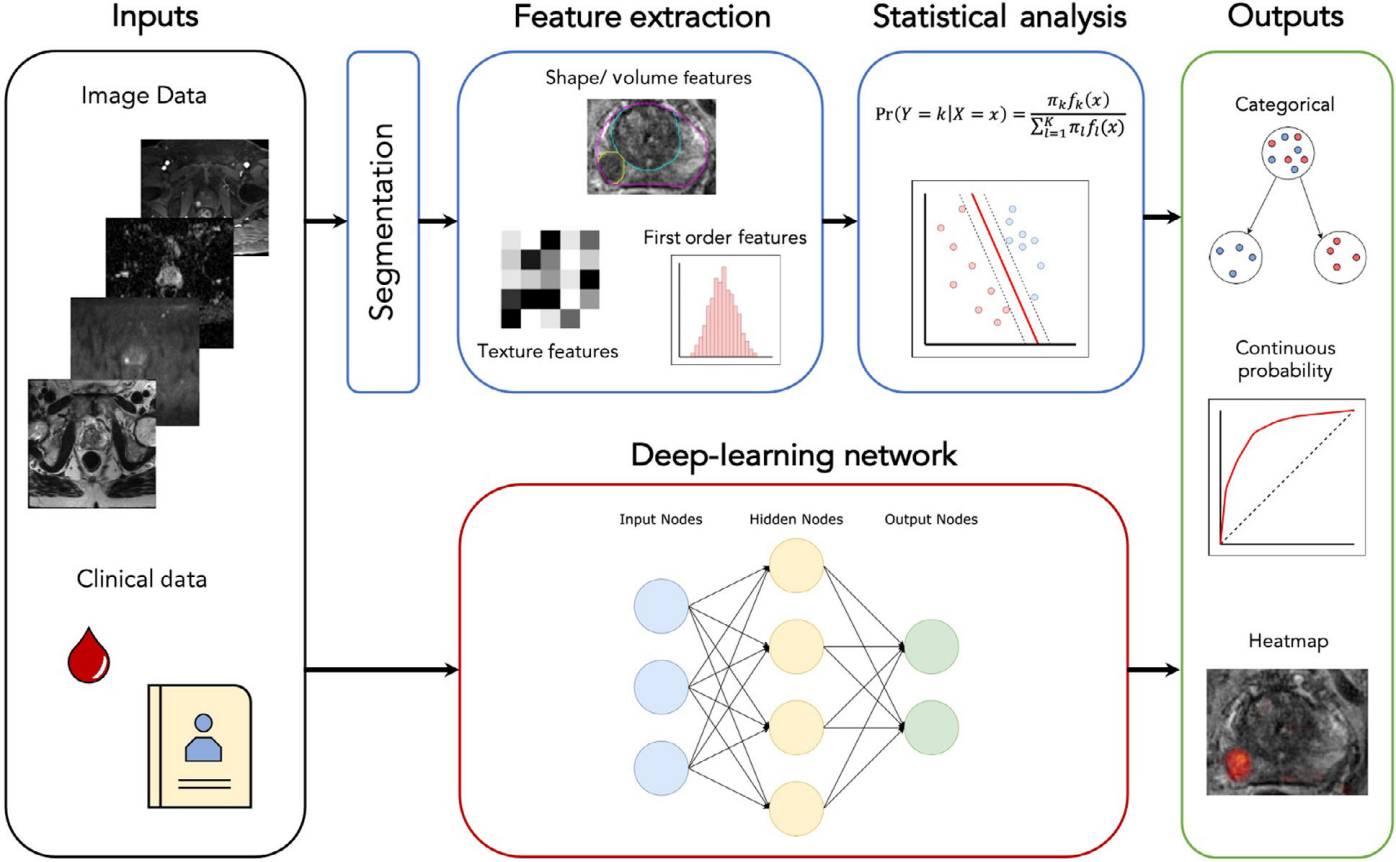
Schematic diagram of common approaches to ML in medical imaging, from left to right. Inputs (black box) often include imaging DICOM (digital imaging and communications in medicine) data and clinical data. For classical ML (blue boxes), images are often segmented and radiomic features extracted for input into the chosen statistical analysis, whereas, for DL methods (red box), the neuronal network uses often unknown discriminating features found during training to classify patients into a specified output (green box), which could be a categorical group or a continuous probability where a receiver operating curve may be produced and, if analysis is done on a voxel‐by‐voxel basis, a heatmap can be constructed.

Deep learning, however, uses networks that in some way resemble neurons of the human brain, comprising multiple layers of interconnected artificial neurons. Each neuron acts as a simple classifier that gives an output based on the inputs from preceding neurons; often, there are a number of layers and hundreds of thousands of individual neurons in a single network (Fig. 1). During the training phase, these DL systems will automatically learn discriminating features without the need for an expert to manually segment tumours; however, much larger training sets are generally required. The output from these methods could be ordinal, such as cancer verses noncancer, or on a continuous scale, predicting the probability of cancer between zero and one, for example. Furthermore, the result could be applied to the whole image, a specified ROI or on a per‐voxel basis. When an ML system is used by a radiologist to aid in interpretation, this is often called computer‐aided diagnosis (CAD).

As an exemplar, in the case of Mp‐MRI of prostate, for which there has been considerable recent interest in ML, multiple CAD systems have been evaluated across various settings. Some systems, which produce probability heatmaps that can be overlaid onto the MRI images and highlight areas of suspicion, have produced stand‐alone performances equivalent to experienced radiologists [55, 56]. Others, however, have demonstrated that, when radiologists use such heatmaps during reporting, they improved their sensitivity for clinically significant cancer by up to 10%, whilst maintaining specificity [57, 58]. Similar systems have also decreased the interobserver variability between radiologists of varying experience [57, 59]. Alternative CAD systems have been designed to improve the classification of lesions in order to reduce false positives and unnecessary biopsies, which is common for indeterminate lesions. Multiple studies have shown that classical ML methods can better characterise suspicious lesions than radiologists [60, 61, 62], with Dinh *et al*. [62] significantly improving per‐lesion specificity by 30% for detecting Gleason 3 + 4 cancer in 129 patients.

At this stage, these positive results are largely limited in generalisability due to similarities in their training and test data sets. For example, we know prostate Mp‐MRI images from different institutions and scanner vendors can look markedly different; therefore, CAD systems need to be trained with large amounts of heterogeneous data in order to be robust enough for widespread clinical use. Gaur *et al*. [59] used a truly external and heterogeneous test set across five institutions and three MR vendors, demonstrating good performance, with the addition of their CAD system similar to with radiologists alone. This proves that CAD systems have the potential to be generalisable, but practical obstacles in obtaining large amounts of well‐labelled heterogeneous data have limited progress in this area. Subsequently, these systems will need to be prospectively assessed for important patient outcomes before CAD becomes part of the diagnostic pathway. Furthermore, DL and ML methods are still often viewed as ‘black boxes’ by clinicians, due to the difficultly in understanding how these techniques arrive at their conclusions [53]. The situation described for PCa imaging is replicated in the field of other cancers, for example breast and colon [63, 64, 65]. These imaging features can be further combined with genetic and clinical information and, together, can perform optimally [66].

### 4.2 Microstructural imaging with tissue model focused MRI

Clinically available microstructural MRI of cancer is reliant on a simple DWI [29], as described above. This conventional method is commonly applied to a range of cancer and noncancer applications (e.g. assessment of inflammatory changes in the bowel [67, 68]) and, as such, is not tissue specific, with multiple pathological mechanisms contributing to signal change. Consequently, whilst standard DWI is an improvement over anatomical imaging alone, it still lacks specificity [69]. One approach to address this is through development of ‘intelligent MRI’; here, unlike the application of a general MRI technique to assess multiple pathologies, individual MRI techniques are crafted to gather specific signals from tissue that are then fed into mathematical models of cancer, in theory allowing a more nuanced assessment of the tissue microstructure [70]. Two such examples, both initially developed for PCa imaging, are highlighted here.

#### 4.2.1 Vascular, extracellular and restricted diffusion for cytometry in tumours MRI

Vascular, extracellular and restricted diffusion for cytometry in tumours (VERDICT) is an imaging technique that builds upon conventional DWI. Initially developed for PCa assessment, VERDICT uses a mathematical model that has three main tissue compartments [71]. These compartments describe the diffusion signal in three separate populations: water molecules trapped inside cells, in the interstitium and inside blood vessels. Similar to conventional DWI, VERDICT MRI acquisition uses clinically available MRI scanners to acquire images with differing b‐values (up to b3000 s·mm^2^), the specific b‐values having been selected from more detailed prior acquisitions to maximise sensitivity to histopathological changes induced by cancer balanced against maintaining a clinically feasible scan time [72]. The acquired data are then fitted to the mathematical model to generate microarchitectural parameters such as intracellular volume fraction (water inside cells), vascular fraction (water inside vessels) and extracellular extravascular fraction (water in the interstitium). Furthermore, estimates of cell radius can also be obtained from this technique.

Similar to many biomarkers, VERDICT MRI was first investigated in animal models. A murine xenograft model of colorectal cancer underwent VERDICT MRI before and after treatment with a chemotherapeutic agent. The study showed that VERDICT MRI could identify significant changes in cell size and vasculature after the administration of a chemotherapeutic agent, in contrast to standard ADC and other diffusion models [71].

Following on from animal work, the technique was tested in eight patients with biopsy‐positive PCa. This feasibility study showed that VERDICT could distinguish between benign and cancerous regions found on biopsy [73]. To obtain further histopathological validation, VERDICT MRI was studied in five patients undergoing prostatectomy. This study showed a strong correlation between VERDICT microstructural parameters such as cellular fraction and collagen fibre patterns in the interstitium to histopathological parameters derived from *ex‐vivo* prostatectomy specimens [74].

Based on the findings from these early studies, VERDICT MRI is being evaluated in a larger cohort of patients as part of the INNOVATE trial [75], a prospective single‐centre study in 365 men suspected of having PCa who undergo VERDICT MRI before biopsy. The aim is to investigate whether VERDICT MRI can improve specificity of clinical Mp‐MRI by reducing the number of indeterminate results. Repeatability and early efficacy of VERDICT MRI was studied in a subset of these patients. This study showed that the VERDICT parameter intracellular fraction (*f*_IC_) was highly repeatable (ICC: 0.87–0.95) and showed potential in differentiating between benign and clinically significant cancer better than ADC [72]. The results of the full trial are awaited.

**Fig. 2:**
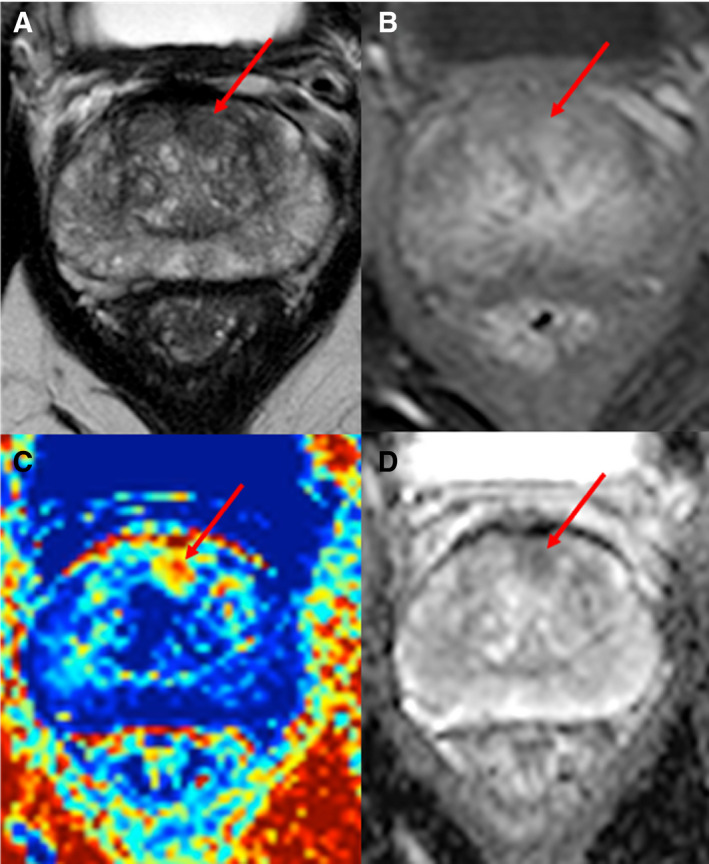
MRI images of a 58 years old with biopsy‐proven PCa. (A) An axial T2‐weighted MRI image showing a focus of homogeneous low signal (red arrow) in the left anterior para‐midline transition zone. (B) T1‐weighted, fat‐suppressed, postcontrast image showing focal enhancement (red arrow) in the left anterior transition zone. (C) VERDICT intracellular volume fraction (FIC) map showing a focal area with increased FIC (red arrow), which enables clearer definition of the biopsy‐positive tumour. (D) ADC map showing reduced signal intensity in the tumour (red arrow).

Outside of the prostate, VERDICT MRI has also shown promise in the characterisation of brain gliomas and bone metastases compared with existing DWI [76, 77]. As an imaging biomarker, it is in the early clinical validation phase, and further work is needed to establish multicentre reproducibility and clinical impact (Fig. 2).

#### 4.2.2 Luminal water fraction imaging

**Fig. 3:**
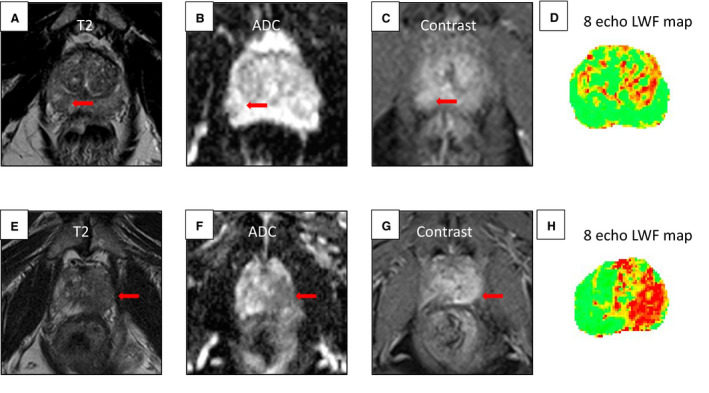
Comparison of conspicuous standard MRI lesions with luminal water fraction (LWF) imaging mapping and targeted biopsy. Lesion indicated by red arrow. Top row from left to right: 65‐year‐old man with PSA 9.9: (A) T2‐weighted MRI showing a region of lesion with reduced signal intensity right periphery. (B) ADC map showing reduced signal intensity in the lesion. (C) Dynamic contrast‐enhanced (DCE) image showing increased signal intensity at the lesion. A Likert score of 4 was given. (D) LWF imaging mapping, which does not indicate the presence of a lesion. Biopsy result: benign. Bottom row from left to right: 68‐year‐old man with PSA 14: (E) T2‐weighted MRI showing a region of reduced signal in the left peripheral zone extending to the transitional zone. (F) ADC mapping, showing reduced signal intensity at the peripheral lesion. (G) DCE imaging, showing lesion enhancement. A Likert score of 5 was given. (H) LWF mapping was positive at the lesion. Biopsy result: positive for cancer Gleason 4 + 3.

Luminal water fraction imaging (LWF) imaging is an MRI technique that has also been developed within the setting of PCa. Here, the mathematical tissue model distinguishes the luminal space of prostate tissue from the stroma and epithelia. In contrast to VERDICT MRI, which is based on DWI, LWF MRI requires multiple T2‐weighted images at differing echo times to populate signals into the model [78]. In pure water, T2 relaxation (the predominant process through which MRI signal decays on T2‐weighted images) is mono‐exponential. However, in the case of prostate tissue, T2 relaxation from fluid contained within microstructural ducts and acini decays significantly more slowly that from the water contained within the surrounding stroma [79], forming two distinct Gaussian distributions of T2 relaxation times. Signal from multiple increasingly T2‐weighted images is input into a mathematical model that separates these two distributions [80] into T‐long, corresponding to the glandular/intraluminal region, and T‐short, which corresponds to the cellular/extraluminal region. An LWF map can then be derived as a fraction volume of luminal space within the tissue (T‐long/(T‐long + T‐short) component [78]). As luminal space is reduced in PCa, with loss of luminal space correlated with increased Gleason tumour grade, LWF MRI has merit for both cancer detection and characterisation (Fig. 3). Initially requiring the acquisition of 64 different signals to calculate, a simplified version utilising a reduced number of T2‐weighted signals has been shown to perform well for cancer detection and characterisation [81].

Luminal water fraction imaging is relatively early in its developmental phase as compared to VERDICT MRI, but its reliance on T2‐weighted imaging has the potential advantage of avoiding common artefacts that degrade image quality of DWI‐based techniques. Currently, however, LWF studies are all single‐centre studies within PCa, with a limited number of subjects; however, the initial clinical validation in these studies shows promise [81]. With ongoing development, LWF could also be applied to the assessment of other glandular organs (e.g. breast and pancreas) for cancer evaluation.

### 4.3 Emerging techniques for metabolic assessment of cancer

There are a number of opportunities to assess cancer metabolism. Novel PET tracers have the potential to provide specificity and exquisite sensitivity to disease [82], whilst new spectroscopic MRI methods utilising hyperpolarised ^13^C‐labelled substrates pave the way to dynamic assessment of tumour metabolism [83].

#### 4.3.1 PET imaging tracers

Fludeoxyglucose (^18^F) is the most widely used tracer in PET‐based imaging and has shown utility in multiple cancer types at different stages of management. In the case of non‐small‐cell lung cancer, FDG‐PET/CT has shown an improvement in staging when compared to CT alone, and utility as a significant prognostication marker, which ultimately leads to changes in clinical management [84, 85, 86]. However, for some cancers, the utility of FDG as an indicator of increased cellular metabolism has limited indications because of inherent biological features of the cancer or because of technical limitations, such as in the case of PCa and renal cell carcinomas, among others [87, 88, 89, 90]. Therefore, multiple tracers have been developed in order to assess other metabolic pathways in cancer and other pathologies.

Choline is one such tracer that exploits the increased cell membrane turnover of malignant cells, resulting in increased choline uptake to be used as a precursor for the biosynthesis of phospholipids [91, 92, 93]. Choline tracing has been largely used in PCa and is found to have a sensitivity and specificity of 85.6% and 92.6%, respectively, for all metastatic sites, and is thus advocated for use in the biochemical recurrence setting [94]. Furthermore, potential utility of choline PET has been investigated in multiple myeloma and hepatocellular carcinoma and has shown promise [95, 96].

Tracers that currently show great promise largely in the setting of biochemical recurrence of PCa but also potentially in the initial staging of higher risk cancers are prostate‐specific membrane antigen (PSMA) and fluorine‐18(^18^F)‐fluciclovine. Fluciclovine is an amino acid analogue that is taken up in greater quantities by PCa cells when compared to surrounding normal tissue, and has been shown to be effective in the early detection of nodal and distant metastasis in recurrent prostate disease, detecting lesions in 57% of patients (122/213) with biochemical recurrence that had equivocal or negative standard of care imaging (abdominopelvic CT or MRI and bone scintigraphy) [97].

Instead, PSMA is a receptor on the cell surface of the prostate cells and increases in density with higher‐grade tumours, metastasis and hormone‐refractory PCa. The precise function is not known, and it is also detected in smaller levels in the small intestine and brain [98, 99]. PSMA PET is characterised by having exceptional node metastasis detection specificities, reaching 99%, significantly outperforming other PET‐CT techniques, and is furthermore not limited to size criteria [100]. Additionally, PSMA PET/CT has greater sensitivities in the detection of bone metastasis when compared to whole‐body bone scans, in one study doubling the number of bone metastases detected [101]. Most significantly, in the setting of biochemical recurrence following radical prostatectomy, PSMA PET/CT showed a detection efficacy of 96.8% for PSA values > 2 and, notably, detection rates of 67% for PSA values < 1, which is markedly better than choline‐based PET/CT, with rates ranging from 19% to 36% [102]. Therefore, PSMA PET/CT has shown utility most significantly in biochemical recurrence. However, there are some limitations, as it is not funded by many healthcare providers and it has been shown that, in very advanced disease, PSMA expression may be lost [103].

#### 4.3.2 Hyperpolarised MRI

**Fig. 4:**
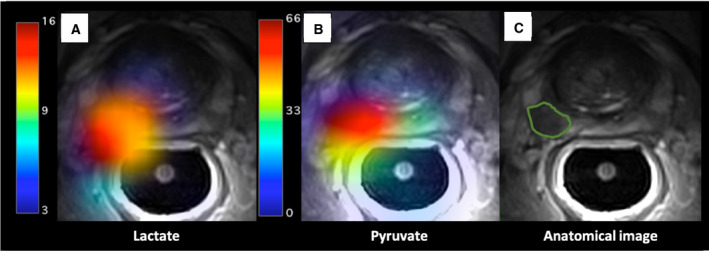
Hyperpolarised 1‐^13^C‐pyruvate‐MRI metabolite maps in MRI‐ and biopsy‐positive PCa. Axial T2‐weighted ^1^H‐MRI scan of a subject with right‐sided, posterior, biopsy‐confirmed Gleason 4 + 3 PCa, segmented in a green line in panel C. Images were acquired with a receive‐only endorectal coil (RAPID Biomedical). Maps of 1‐^13^C‐pyruvate (centre, panel B) and 1‐^13^C‐lactate (left, panel A) were produced, via an IDEAL model, from multiecho‐bSSFP data (13C‐MRI) and overlaid on the original T2W acquisition; demonstrating the distribution of hyperpolarised 1‐^13^C‐pyruvate (panel B) and its downstream metabolites, (lactate in panel A) 25 s after contrast injection.

Carbon serves as the backbone of nearly all organic molecules, and MRI signal can be generated from ^13^C nuclei, making carbon an attractive target for imaging. However, ^13^C imaging remains technically challenging. First, the natural abundance of ^13^C is only 1.1% when compared to 99% for ^1^H, which is used to generate signal for conventional MRI (Fig. 4). Therefore, ^13^C probes are synthetically enriched to increase the concentration of ^13^C, typically to 99%, and, through the process of hyperpolarisation, the MR signal of ^13^C‐labelled substrates is boosted by a factor >10 000 [104]. This typically allows 35–40 mL of hyperpolarised substrate in solution to provide sufficient signal following intravenous injection [105]. Once the dissolute is ready for injection, the hyperpolarised state (and hence signal) for 1‐^13^C‐pyruvate decays rapidly, with a half‐life of 45–60 s [104, 106, 107]. Injection and imaging must therefore be rapidly completed, ideally within 2–3 min. This poses a number of technical and logistical challenges [83], limiting the current use of hyperpolarised methods to research within specialised centres.

Whilst a number of hyperpolarised substrates are available in the preclinical setting [108], to date only hyperpolarised ^13^C‐labelled pyruvate has been trialled in humans [109, 110, 111, 112, 113]. Pyruvate is the end product of glycolysis and can be converted to lactate via the enzyme lactate dehydrogenase or to alanine by alanine transaminase. Pyruvate can also be transported into the mitochondria, where it is converted to acetyl‐CoA CO_2_ by pyruvate dehydrogenase. As such, following injection of hyperpolarised 1‐^13^C‐pyruvate, an MRI signal is typically observed from lactate, alanine and/or bicarbonate dependent on tissue and pathology [114]. Typically, imaging is performed with a temporal resolution of 3–6 s, allowing for dynamic changes in the strengths of signals from pyruvate and its downstream products to be documented, and thereby for enzymatic fluxes to be interrogated [107, 113].

Human dose escalation and safety study of hyperpolarised 1‐^13^C‐pyruvate were reported in 2013 [105]. Since then, there have been a number of reported first‐in‐human clinical applications, including prostate, renal and breast cancer [105, 115, 116]. Immunohistochemical analysis of tumour regions detected with 1‐^13^C‐pyruvate hyperpolarised MRI (HP‐MRI) confirms an overexpression of monocarboxylate transport 1, which imports pyruvate and lactate into cells [112]. As well as detection and potential evaluation of disease aggressiveness of PCa [112, 117], 1‐^13^C‐pyruvate HP‐MRI has also demonstrated proof of concept as a metabolic response biomarker to androgen‐deprivation therapy [113].

HP‐MRI has the potential to provide nonradioactive and unique‐in‐human dynamic assessment of tumour metabolism, providing opportunities for tumour detection, characterisation and treatment response assessment. Currently, it is in the very early stages of development and will likely need: (a) advances in hyperpolariser hardware to reduce cost and complexity [118] and (b) implementation of methods to prolong the hyperpolarised state [119] if it is to enter widespread clinical use.

## 5 Conclusion

Major technological advances have been made in the last 2 decades that have provided new radiological tools with which to interrogate cancer. Anatomical imaging remains the mainstay for the assessment of disease and treatment response [120, 121], but new clinically available MRI techniques that can evaluate tumour cellularity and vascularity are now commonly utilised for selected tumours, with PCa being used in this exemplar [14, 122, 123]. Imaging can yet do more, and new research is emerging on methods that improve the microstructural detail that imaging can depict [70] together with moving into the assessment of metabolic processes, in some cases in real time [124]. Through careful and methodological development, these techniques may provide clinicians more sensitive and specific markers for cancer assessment.

## Conflict of interest

The authors declare no conflict of interest.

## Author contributions

AR first author of manuscript. FG, TS, SS and SA contributed to manuscript including image and diagram development. SP conceived, supervised, edited and contributed to manuscript.
